# Analysis of physical mechanisms for channel-length-dependent PBTS reliability in SA TG coplanar IGZO TFTs

**DOI:** 10.1038/s41598-025-27549-x

**Published:** 2025-12-10

**Authors:** Dong-Hwi Son, Chae-Eun Oh, Hyeon-Woo Lee, Chan-Yong Jeong, Jae-Man Jang, Byung-Du Ahn, Jong-Uk Bae, Hyuck-In Kwon

**Affiliations:** 1 Major in Intelligent Semiconductor Engineering, Chung-Ang University, 84 Heukseok-ro, Dongjak-gu, Seoul, Korea; 2 Research and Development Center, LG Display Company, Paju, 10845 Korea

**Keywords:** SA TG coplanar IGZO TFTs, Channel length, PBTS reliability, Subgap DOS, Near-interface gate dielectric trap density, Hydrogen diffusion, Engineering, Materials science, Nanoscience and technology, Physics

## Abstract

This study investigates the physical mechanisms for channel-length-dependent positive bias temperature stress (PBTS) reliability in self-aligned top-gate (SA TG) coplanar indium-gallium-zinc oxide (IGZO) thin-film transistors (TFTs). We fabricated devices with channel lengths of 3 µm, 12 µm, and 20 µm and characterized them using high-low frequency capacitance-voltage measurements and low-frequency noise analysis. Experimental results show that the 3 µm channel length device exhibits a significantly lower subgap density of states in the IGZO channel and a reduced near-interface trap density in the gate dielectric compared to its longer-channel counterparts. These reductions are strongly correlated with the enhanced PBTS reliability of the short-channel SA TG coplanar IGZO TFTs. We propose that hydrogen diffusion from the n^+^-IGZO source/drain extensions during fabrication may be the underlying mechanism, leading to defect passivation in both the IGZO channel and the SiO_2_ gate dielectric. These findings offer physical insights into the degradation behavior of IGZO TFTs and provide practical guidance for designing highly reliable backplane transistors for advanced active-matrix organic light-emitting diode displays.

## Introduction

Indium-gallium-zinc oxide (IGZO) has emerged as a promising alternative to conventional amorphous silicon (a-Si) for thin-film transistor (TFT) applications, owing to its superior carrier mobility, compatibility with low-temperature processing, and low off-state current^1–3^. As a result, IGZO TFTs have been widely employed in commercial displays, particularly in active-matrix organic light-emitting diode (AMOLED) technologies^4–6^. Among the various IGZO TFT architectures, the self-aligned top-gate (SA TG) coplanar structure has been widely adopted for AMOLED backplanes, offering advantages such as low parasitic capacitance and enhanced process controllability^7,8^. As AMOLED displays push toward higher pixel densities, scaling the channel length of TFTs has become a key design requirement. However, it has been reported that in SA TG coplanar IGZO TFTs, both the electrical characteristics and long-term reliability can vary significantly with channel length^9–12^. In particular, several studies have shown that positive bias temperature stress (PBTS) reliability tends to improve as the channel length decreases^13–15^. This phenomenon has been discussed in previous studies with several hypotheses, but a clear physical explanation supported by experimental validation remains insufficient.

In this study, we investigated the origin of the channel-length-dependent PBTS behavior in SA TG coplanar IGZO TFTs. Devices with different channel lengths were characterized using high-low frequency capacitance-voltage (*C-V*) measurements and low-frequency noise (LFN) techniques. These methods allow for the quantitative extraction of the subgap density of states (DOS) in the IGZO channel and the near-interface trap density in the gate dielectric, both of which are key contributors to PBTS-induced degradation in IGZO TFTs. Our results indicate that the improved PBTS reliability in short-channel devices is associated with concurrent reductions in both the subgap DOS of the IGZO channel and the near-interface trap density in the gate dielectric. These findings provide physical insight into the channel-length-dependent degradation behavior in SA TG coplanar IGZO TFTs and offer guidance for the design of highly reliable IGZO TFTs for advanced display applications.

## Result and discussion

**Fig. 1:**
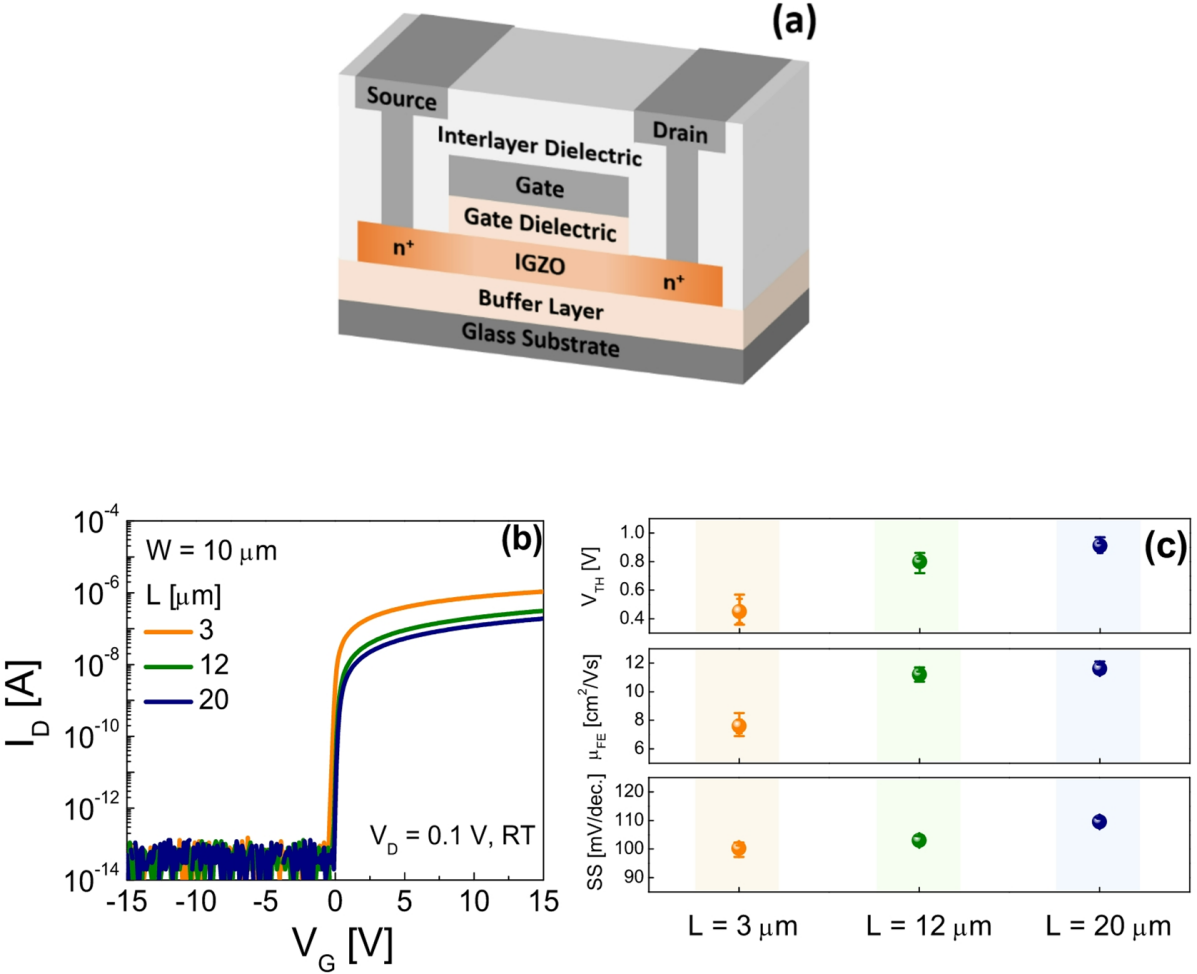
(**a**) Schematic cross-sectional of the fabricated SA TG coplanar IGZO TFTs. (**b**) Transfer characteristics of the SA TG coplanar IGZO TFTs with various *L*s (*W*/*L* = 10 µm/3, 12, 20 µm) measured in the linear region (*V*_D_ = 0.1 V) at RT. (**c**) *V*_TH_, *µ*_FE_, and *SS* values extracted from the SA TG coplanar IGZO TFTs with various *L*s (*W*/*L* = 10 µm/3, 12, 20 µm), where each value represents the average of five devices, and the vertical error bars indicate ± 1 standard deviation.

Figure 1a presents a schematic cross-sectional view of the fabricated SA TG coplanar IGZO TFTs. The detailed fabrication procedure is provided in the Methods section. Figure 1b shows the transfer characteristics of devices with channel lengths (*L*) of 3 µm, 12 µm, and 20 µm, with a fixed channel width (*W*) of 10 µm, measured at room temperature (RT). During the measurements, the gate voltage (*V*_G_) was swept from − 15 V to + 15 V, while the drain voltage (*V*_D_) was held constant at 0.1 V, and the corresponding drain current (*I*_D_) was recorded. All electrical measurements were conducted using an Agilent 4156 C semiconductor parameter analyzer. Figure 1c summarizes the electrical parameters of SA TG coplanar IGZO TFTs with various *L*s(*W*/*L* = 10 µm/3, 12, 20 µm). Each point is the mean of five devices for a given channel length, and the vertical error bars denote ± 1 standard deviation. The threshold voltage *V*_TH_ was extracted as the gate voltage *V*_G_ corresponding to *I*_D_ = (*W*/*L*) × 1 nA. As the channel becomes shorter, *V*_TH_ decreases, which is attributed to hydrogen diffusion from the n^+^-IGZO source/drain extension regions into the channel, increasing the carrier concentration^16^. The field-effect mobility *µ*_FE_ denotes the apparent mobility, extracted without considering the source/drain parasitic resistance *R*_ext_. It was computed in the linear region at *V*_D_ = 0.1 V using *µ*_FE_ = *g*_m_*L*/*WC*_OX_*V*_D_, where *g*_m_ is the transconductance and *C*_OX_ is the gate dielectric per unit area, and the reported value corresponds to the maximum extracted *µ*_FE_. Consequently, shorter channels, for which the relative *R*_ext_ is larger, exhibit a smaller extracted *µ*_FE_ despite their higher carrier concentration. The subthreshold swing *SS* was calculated as (dlog(*I*_D_)/d*V*_G_)^−1^ in the *I*_D_ range of 10^− 11^ – 10^− 10^ A and decreases as *L* becomes shorter.

**Fig. 2:**
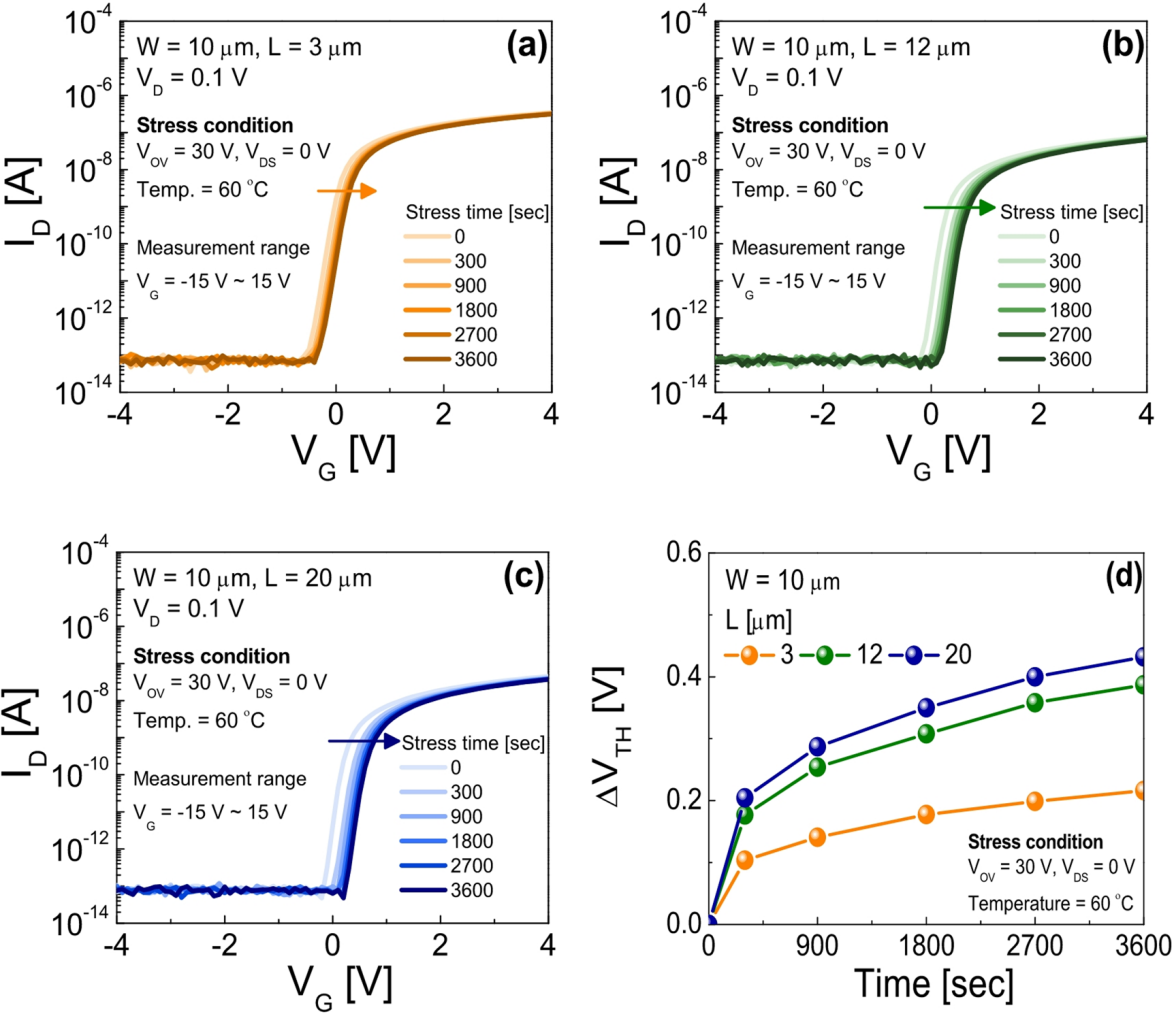
Time evoluition of the transfer characteristics for SA TG coplanar IGZO TFTs with channel lengths of (**a**) 3 µm, (**b**) 12 µm, and (**c**) 20 µm under PBTS of *V*_OV_ = 30 V and *V*_S_ = *V*_D_ = 0 V at 60 ℃. (**d**) Δ*V*_TH_ values under PBTS for IGZO TFTs with different channel lengths as a function of stress time.

Figure 2a–c display the time evolution of the transfer characteristics for SA TG coplanar IGZO TFTs with channel lengths of (a) 3 µm, (b) 12 µm, and (c) 20 µm under PBTS conditions: overdrive voltage *V*_OV_ (= *V*_G_ - *V*_TH_) = 30 V, *V*_S_ = *V*_D_ = 0 V, and temperature = 60 ℃, where *V*_S_ denotes the source voltage. Figure 2d summarizes the threshold voltage shift (Δ*V*_TH_) under PBTS for devices with different channel lengths as a function of stress time. From Fig. 2, it is observed that *V*_TH_ increases with stress time in all devices: however, the Δ*V*_TH_ values decrease as the channel length becomes shorter. This trend is consistent with previous reports indicating that short-channel SA TG coplanar IGZO TFTs exhibit improved PBTS stability compared to their long-channel counterparts^13–15^. Furthermore, the results in Fig. 2 clearly show that the device with a 3 µm channel length demonstrates particularly enhanced PBTS reliability relative to the devices with channel lengths of 12 µm and 20 µm.

**Fig. 3:**
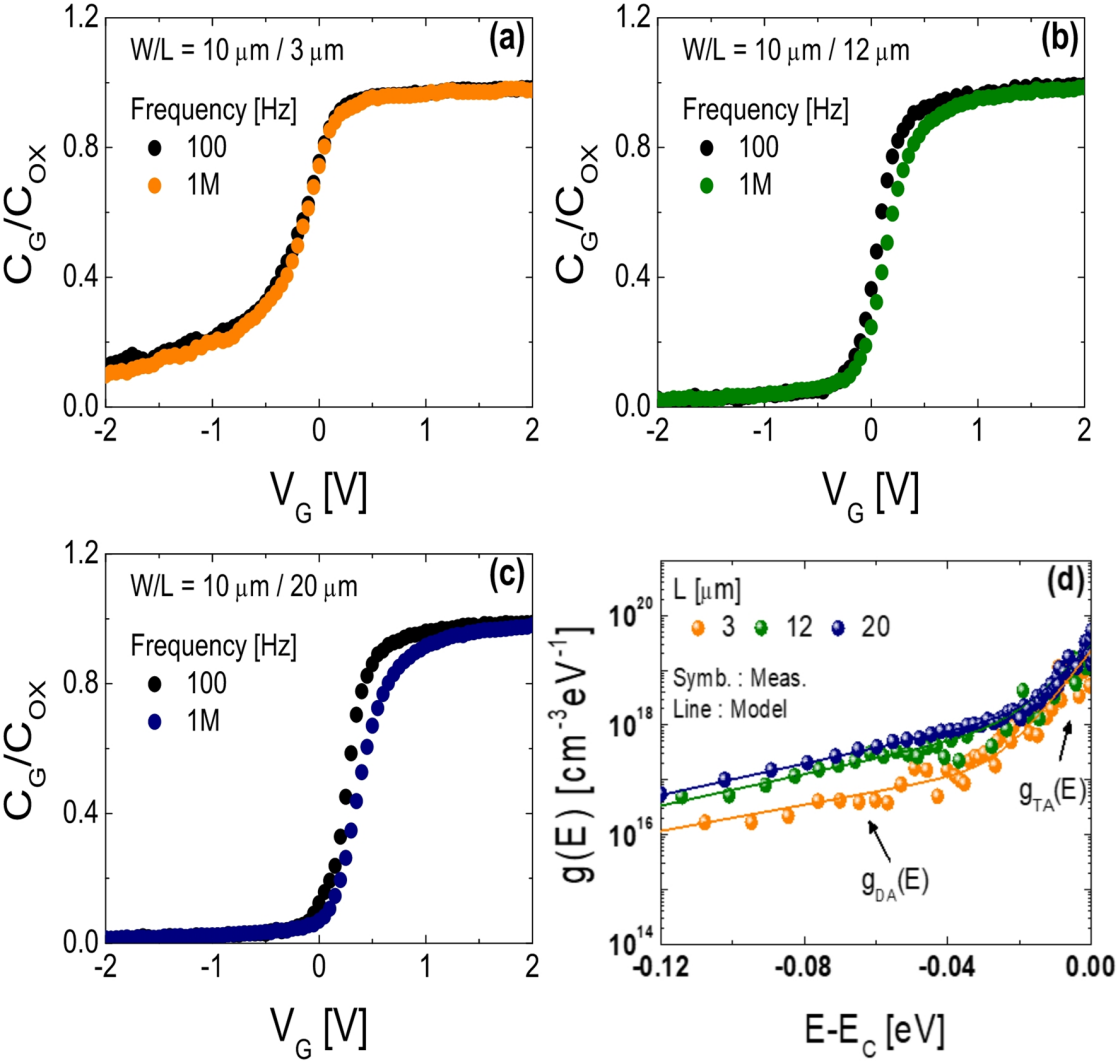
Normalized *C*_G_-*V*_G_ (*C*_G_/*C*_OX_-*V*_G_) curves measured at low (*f* = 100 Hz) and high (*f* = 1 MHz) frequencies for SA TG coplanar IGZO TFTs with channel lengths of (**a**) 3 µm, (**b**) 12 µm and (**c**) 20 µm. (**d**) Energy distribution of subgap DOS value (*g*(E)) extracted from the fabricated SA TG coplanar IGZO TFTs with *L* = 3 µm, 12 µm, and 20 µm near *E*_C_.

To elucidate the physical mechanisms responsible for the phenomena observed in Fig. 2, we characterized the fabricated SA TG coplanar IGZO TFTs with various channel lengths using high-low frequency (*f*) *C-V* measurements and LFN techniques. Figure 3a–c present the normalized *C*_G_-*V*_G_ (*C*_G_/*C*_OX_-*V*_G_) curves measured at low (*f* = 100 Hz) and high (*f* = 1 MHz) frequencies for IGZO TFTs with *L* = (a) 3 µm, (b) 12 µm, (c) 20 µm, respectively. Here, *C*_G_ denotes the gate-to-channel capacitance per unit area measured between the gate and the source/drain electrodes. *C-V* measurements were performed using a Solartron SI 1260 impedance analyzer coupled with an SI 1296 dielectric interface module. The results in Fig. 3a–c clearly demonstrate that the frequency-dependent dispersion of the normalized *C*_G_-*V*_G_ characteristics becomes more pronounced with increasing channel length. In IGZO TFTs, the capacitance per unit area induced by localized subgap states (*C*_LOC_) can be extracted using the high-low frequency *C-V* method, as shown in Eq. (1)^17,18^, and the subgap DOS (*g*(E)) can be calculated using Eq. (2)^19^.

1 $${C_{LOC}}\,\,=\,\,\,\,\left[ {\,\,{{\left( {\frac{1}{{{C_{LF}}}}\,\, - \,\,\frac{1}{{{C_{OX}}}}} \right)}^{ - 1}}\,\, - \,\,{{\left( {\frac{1}{{{C_{HF}}}}\,\, - \,\,\frac{1}{{{C_{OX}}}}} \right)}^{ - 1}}\,\,} \right]\,\,\,\,\,[F/c{m^{ - 2}}]$$

2 $$g\,(E)\,\,=\,\,\frac{{{C_{LOC}}}}{{{q^2}{t_{IGZO}}}}\,\,\,\,\,[c{m^{ - 3}}e{V^{ - 1}}]$$

Here, *C*_LF_ and *C*_HF_ denote the gate-to-channel capacitance per unit area measured at low and high frequencies, respectively; *t*_IGZO_ is the channel thickness of the TFTs; and *q* is the elementary charge. The surface potential ($\:{\varphi\:}_{s}$) can be nonlinearly mapped to *V*_G_ using Eq. (3)^20^

3 $${\phi _s}({V_G})\;\;=\;\;\int\limits_{{{V_{FB}}}}^{{{V_G}}} {\;\;\left( {1 - \frac{{{C_G}({V_G})}}{{{C_{OX}}}}} \right)} \,\,\,d{V_G}\,\,\,[{\text{V}}]$$

where *V*_FB_ is the flat-band voltage. Figure 3d presents the energy distribution of subgap DOS values extracted from the fabricated SA TG coplanar IGZO TFTs with L = 3 µm, 12 µm, and 20 µm near conduction band minimum (*E*_C_). The extracted subgap DOS values are divided into two components according to their energy level distribution: acceptor-like deep states (*g*_DA_) and acceptor-like tail states (*g*_TA_), in an increasing order of the energy levels. The subgap DOS profiles of each TFT near *E*_C_ are well fitted with the following model

4 $$\begin{gathered} g\,(E)\,\, = \,\,g_{{DA}} \,(E)\,\, + \,\,g_{{TA}} \,(E)\,\, = \,\,N_{{DA}} \,\, \times \,\,\exp \,\,\left( {\frac{{E\,\, - \,\,E_{C} }}{{kT_{{DA}} }}} \right) \hfill \\ \,\,\,\,\, + \,\,N_{{TA}} \,\, \times \,\,\exp \left( {\frac{{E\,\, - \,\,E_{C} }}{{kT_{{TA}} }}} \right)\,\,\,\,[{\text{cm}}^{{ - 3}} {\text{eV}}^{{ - 1}} ] \hfill \\ \end{gathered}$$

where *E* is the electron energy; *N*_DA_ and *N*_TA_ are the densities of acceptor-like deep and tail states, extrapolated to *E*_C_, respectively; and *kT*_DA_ and *kT*_TA_ are the corresponding characteristic energies. Table 1 summarizes the subgap DOS parameters extracted from every TFT. Figure 3d and Table 1 indicate that the subgap DOS decreases with decreasing channel length in the fabricated SA TG IGZO TFTs. In particular, the subgap DOS extracted from the device with *L* = 3 µm is significantly lower than that extracted from the devices with *L* = 12 µm or 20 µm. Previous studies have reported that a high density of acceptor-like states can degrade the PBTS reliability of IGZO TFTs^21–24^. The experimental results in Fig. 3; Table 1 are consistent with those in Fig. 2 and suggest that the lower subgap DOS contributes to the superior PBTS reliability observed in the short-channel SA TG coplanar IGZO TFTs.

**Table 1:** Subgap DOS parameters extracted from the fabricated SA TG IGZO TFTs with different channel lengths (*L* = 3 µm, 12 µm, and 20 µm).

Parameter	L = 3 μm	L = 12 μm	L = 20 μm
*N*_*TA*_ [cm^− 3^eV^− 1^]	2.3 × 10^19^	2.9 × 10^19^	3.0 × 10^19^
*kT*_TA_ [eV]	0.005	0.006	0.005
*N*_DA_ [cm^− 3^eV^− 1^]	3.2 × 10^17^	1.8 × 10^18^	2.8 × 10^18^
*kT*_*DA*_ [eV]	0.036	0.030	0.030

**Fig. 4:**
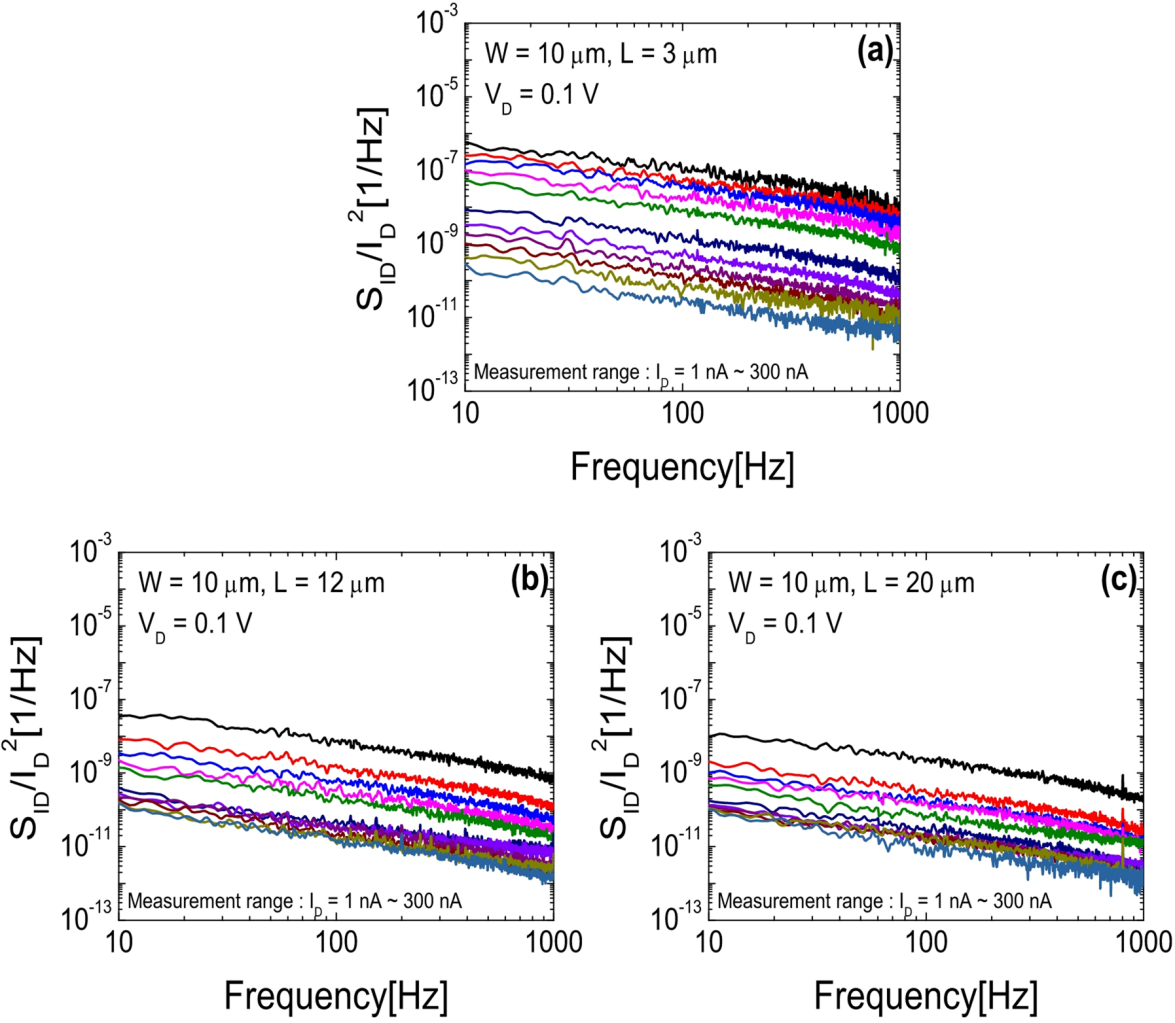
Frequency dependence of *S*_ID_/*I*_D_^2^ values measured at various *I*_D_s for SA TG coplanar IGZO TFTs with channel lengths of (**a**) 3 µm, (**b**) 12 µm, and (**c**) 20 µm.

**Fig. 5:**
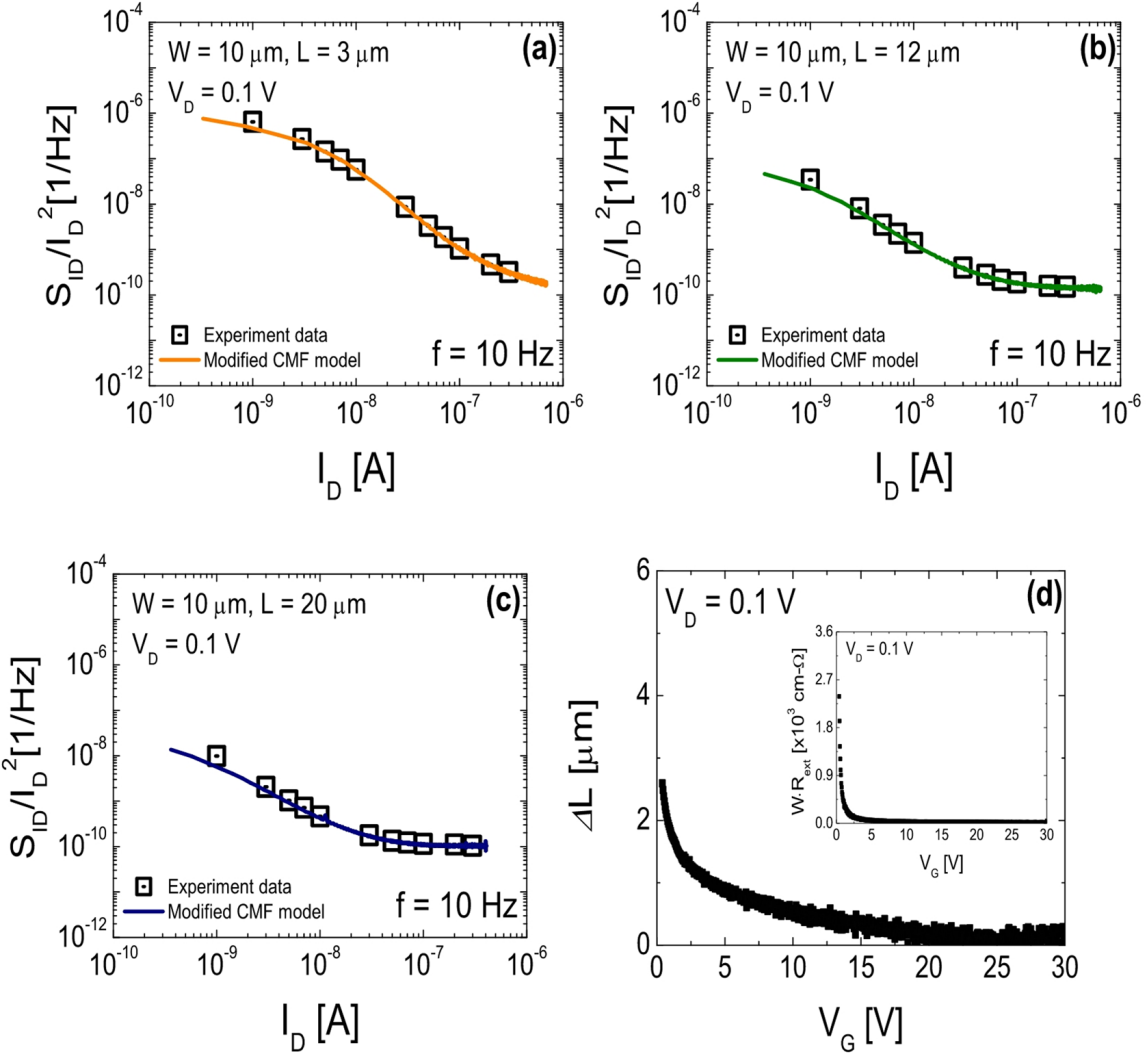
*S*_ID_/*I*_D_^2^ versus *I*_D_ plot for SA TG coplanar IGZO TFTs with channel lengths of (**a**) 3 µm, (**b**) 12 µm, and (**c**) 20 µm. The symbols represent the experimental data, while the solid lines denote the fitting curves obtained using the modified LFN model. (**d**) Δ*L* values as a function of *V*_G_; the inset shows *W·R*_*ext*_ versus *V*_G_, both extracted using the *V*_G_-based transmission line method.

Figure 4a–c show the frequency-dependent behavior of the normalized drain current noise power spectral density (*S*_ID_/*I*_D_^2^), measured at various *I*_D_ levels by sweeping *V*_G_ under a fixed *V*_D_ of 0.1 V, for SA TG coplanar IGZO TFTs with channel lengths of (a) 3 µm, (b) 12 µm, and (c) 20 µm, respectively. LFN characteristics were measured under ambient conditions at RT using an Agilent 89,441 vector signal analyzer and an SR570 low-noise current amplifier (Stanford Research Systems). Figure 5a–c present *S*_ID_/*I*_D_^2^ as a function of *I*_D_ at *f* = 10 Hz for each device. The symbols represent the experimental data, while the solid lines denote the fitting curves obtained using the modified LFN model described in Eq. (5), which was developed by our group to accurately capture the LFN behavior in SA TG coplanar oxide TFTs^25^.

5 $$\frac{{{S_{{I_D}}}}}{{{I_D}^{2}}}={\left( {1+{\alpha _C}{\mu _{eff}}{C_{OX}}\frac{{{I_D}}}{{{g_{mi}}}}} \right)^2}{\left( {\frac{{{R_{Ch}}}}{{{R_{Tot}}}}\frac{{{g_{mi}}}}{{{I_D}}}} \right)^2}\frac{{{q^2}kT\lambda {N_B}}}{{fW{L_{eff}}{C_{OX}}^{2}}}\,\,$$

This model extends the conventional Δ*N*-Δ*µ* framework by incorporating structural effects unique to SA TG architectures-specifically, gate-length modulation (Δ*L*) and large source/drain parasitic resistance (*R*_ext_)-thereby enabling improved extraction of gate dielectric trap parameters in SA TG coplanar IGZO TFTs. Therefore, the quality of the gate dielectric can be evaluated more precisely and quantitatively, and it can be effectively used to analyze the reliability of devices, especially in short-channel structures where the influence of effective channel length and parasitic resistance elements are relatively large. In Eq. (5), *N*_B_ is the near-interface trap density, *k* the Boltzmann constant, *T* the temperature, *g*_mi_ the intrinsic transconductance, *α*_C_ the Coulomb scattering coefficient, *λ* the tunneling attenuation length in the gate dielectric (= 10^− 8^ cm)^26^, and *R*_Ch_ and *R*_Tot_ the channel and total resistances, respectively. The effective mobility *µ*_eff_ is defined in Eq. (6), which accounts for the effective channel length (*L*_eff_ = *L* - Δ*L*) and *R*_ext_ extracted from the fabricated devices.

6 $${\mu _{eff}}=\frac{1}{{{C_{OX}}\left( {{V_D} - 2{I_D}{\operatorname{R} _{ext}}} \right)}}\frac{{{L_{eff}}}}{W}\frac{{\partial {I_D}}}{{\partial \left( {{V_G} - {I_D}{\operatorname{R} _{ext}}} \right)}}\,\,\,$$

**Fig. 6:**
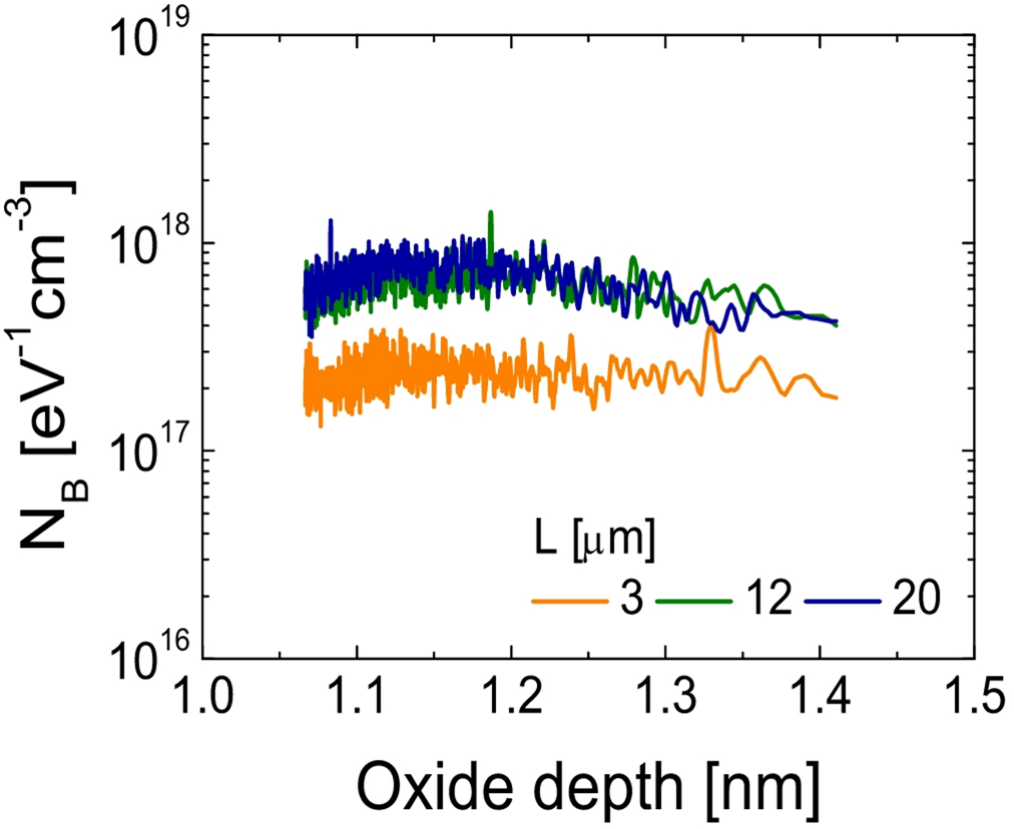
Spatial distribution of *N*_B_s with respect to the distance from the IGZO/SiO_2_ interface in SA TG coplanar IGZO TFTs with channel lengths of (**a**) 3 µm, (**b**) 12 µm, and (**c**) 20 µm.

Figure 5d and the inset present the extracted Δ*L* and *W·R*_ext_ as functions of *V*_G_, obtained using the paired *V*_G_-based transmission line method at *V*_D_ = 0.1 V^27^. Figure 6 illustrates the spatial profiles of *N*_*B*_ as a function of tunneling depth from the IGZO/SiO_2_ interface, where the depth *x* is calculated from frequency via Eq. (7), assuming a characteristic interface time constant *τ*_0_ ≈ 10^− 10^s^28^.

7 $$x=\lambda \cdot \ln (\frac{1}{{2\pi f{\tau _0}}})\,\,$$

As shown in Fig. 6, the extracted *N*_B_ values over the tunneling depth range (*x* ~ 1.1–1.4 nm) show a decreasing trend with shorter channel lengths, and this trend appears more noticeable in the device with *L* = 3 µm compared to those with *L* = 12 µm and 20 µm. Given that enhanced electron trapping into the gate dielectric under PBTS typically results in greater threshold voltage shifts^29,30^, this observation suggests that reduced *N*_B_ near the IGZO/SiO_2_ interface also contributes to the improved PBTS reliability seen in the short-channel SA TG coplanar IGZO TFTs.

**Fig. 7:**
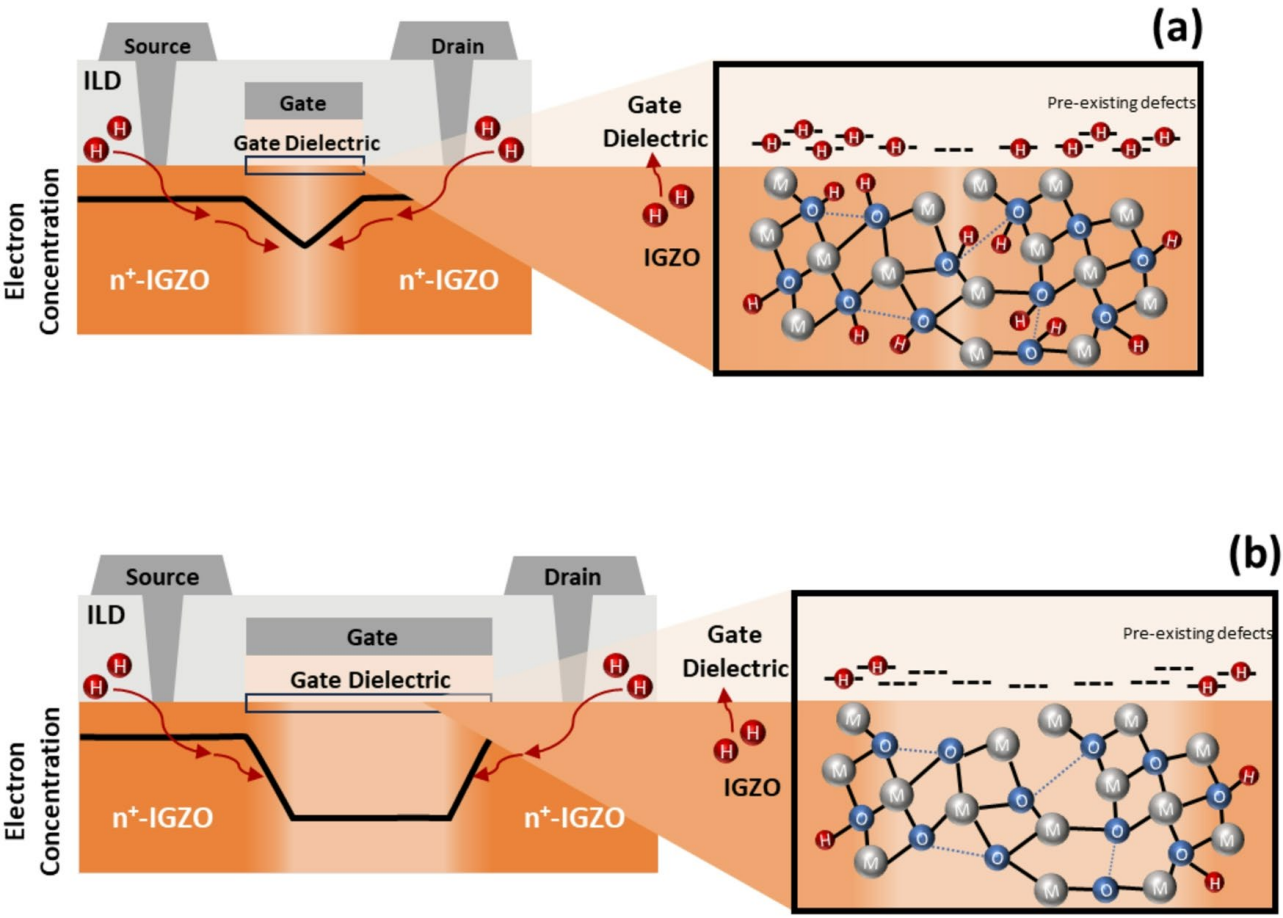
Comparison of the spatial distributions of carrier concentration and hydrogen diffusion effects in (**a**) short-channel (*L* = 3 µm) and (**b**) long-channel (*L* = 12 or 20 µm) SA TG coplanar IGZO TFTs.

The experimental results presented in Figs. 3, 4, 5 and 6 clearly indicate that the enhanced PBTS reliability observed in shorter-channel SA TG coplanar IGZO TFTs is closely related to the simultaneous reduction in both the subgap DOS in the IGZO channel and the near-interface trap density in the SiO_2_ gate dielectric. To account for the observed behavior, we propose the following physical model to explain the underlying mechanism responsible for the improved PBTS reliability in short-channel devices, as illustrated in Fig. 7. In SA TG coplanar IGZO TFTs, the source/drain extension regions are heavily n^+^-doped, primarily due to the diffusion of hydrogen atoms originating from the PECVD-deposited ILD into the IGZO films. The role of hydrogen in IGZO TFTs has been reported to vary depending on its concentration^31,32^. Given that *V*_TH_ and *SS* both decrease as the channel length decreases, this study focused on hydrogen serving dual roles as an electron donor (e.g., H^0^ → H^+^ + e-) and as a defect passivator (e.g., ≡M-O• + H → ≡M-OH)^33–35^. During the post-deposition annealing step, hydrogen diffuses from the n^+^-IGZO extension regions into the adjacent channel region, leading to an increased free electron concentration and suppressed subgap DOS in IGZO^16^. In addition, the elevated hydrogen concentration in the IGZO channel facilitates the subsequent diffusion of hydrogen into the SiO_2_ gate insulator. The incorporated hydrogen atoms can passivate pre-existing defects in SiO_2_, likely via hydrogen-related chemical reactions such as ≡ Si• + H → ≡Si-H^36,37^, thereby reducing the electrical trap density near the IGZO/SiO_2_ interface. Figure 7a and 7(b) schematically compare the spatial distributions of carrier concentration and hydrogen diffusion effects between short-channel (*L* = 3 µm) and long-channel (*L* = 12 or 20 µm) devices. Given that the effective hydrogen diffusion length from the n^+^-IGZO source/drain extension into the channel region is approximately 2.5-3.0 µm, as shown in Fig. 5d, it is plausible that almost the entire channel of the 3 µm device is subject to hydrogen-induced modifications. This may account for the markedly improved PBTS reliability of the shortest-channel device relative to its longer-channel counterparts. However, when the channel length becomes shorter than the hydrogen diffusion length (Δ*L*) from the n^+^-IGZO source/drain extension regions, the carrier concentration in the channel increases excessively, which may lead to abnormal device operation. Therefore, the excellent PBTS tendency for shorter channel devices should only be considered valid within the channel length range where normal operation is guaranteed. However, since Δ*L* in SA TG Coplanar Oxide TFTs varies depending on process conditions such as temperature during the post-deposition process, if normal operation characteristics are secured for devices with submicron-scale channel lengths through process optimization, the phenomenon observed in this study is expected to be valid for the relevant devices as well.

## Conclusion

In this study, we conducted a comprehensive analysis to identify the physical mechanisms responsible for the channel-length-dependent PBTS reliability in SA TG coplanar IGZO TFTs. Our experimental results demonstrated that short-channel devices exhibited superior PBTS reliability, with a significantly reduced Δ*V*_TH_ compared to longer-channel devices. To understand this phenomenon, we employed high-low frequency *C-V* and LFN characterization methods. The *C-V* analysis revealed a clear trend: the subgap DOS in the IGZO channel decreased as the channel length was reduced. Concurrently, LFN measurements showed that the near-interface trap density in the SiO_2_ gate dielectric also decreased with shorter channel lengths. These findings confirm that both the IGZO bulk properties and the quality of the SiO_2_ gate dielectric are improved in shorter-channel devices. We possibly attribute this improvement to hydrogen diffusion from the n^+^-IGZO source/drain extensions during fabrication, which may passivate defects in both regions. In particular, in devices with *L* = 3 µm, almost the entire channel lies within the effective hydrogen diffusion range, leading to a substantial reduction in defect density and, consequently, improved PBTS reliability.

## Methods

The fabrication process commenced with the deposition of a 300 nm-thick SiO_2_ buffer layer on a glass substrate via plasma-enhanced chemical vapor deposition (PECVD). A 30 nm-thick amorphous IGZO film (In: Ga: Zn = 1:1:1 mol%) was subsequently formed at room temperature using DC magnetron sputtering. A 150 nm-thick SiO_2_ gate dielectric was then deposited through PECVD, followed by the formation of a Cu/MoTi gate electrode using DC sputtering. After photolithographic definition of both the gate and gate insulator layers, interlayer dielectric (ILD) films consisting of SiO_x_ and SiN_x_ were sequentially deposited by PECVD and patterned to create contact vias. Source and drain electrodes composed of Cu/MoTi were then deposited and patterned to align with the gate, completing the formation of n^+^-IGZO source/drain extension regions. During this process, hydrogen atoms originating from the PECVD-deposited ILD layers diffused into the IGZO films, donating electrons and thereby modulating its conductivity in the source/drain extension regions. Finally, a top passivation layer of SiO_2_ was deposited over the entire device structure, and thermal annealing was performed to stabilize electrical characteristics and improve film uniformity.

## Data Availability

The datasets used and/or analyzed during the current study available from the corresponding author on reasonable request.
